# A Genomics-Based Model for Prediction of Severe Bioprosthetic Mitral Valve Calcification

**DOI:** 10.3390/ijms17091385

**Published:** 2016-08-31

**Authors:** Anastasia V. Ponasenko, Maria V. Khutornaya, Anton G. Kutikhin, Natalia V. Rutkovskaya, Anna V. Tsepokina, Natalia V. Kondyukova, Arseniy E. Yuzhalin, Leonid S. Barbarash

**Affiliations:** 1 Research Institute for Complex Issues of Cardiovascular Diseases, Sosnovy Boulvevard 6, Kemerovo 650002, Russia; ponaav@kemcardio.ru (A.V.P.); masha_hut@mail.ru (M.V.K.); rutknv@kemcardio.ru (N.V.R.); annacepokina@mail.ru (A.V.T.); kondnv@kemcardio.ru (N.V.K.); arseniy.yuzhalin@oncology.ox.ac.uk (A.E.Y.); reception@kemcardio.ru (L.S.B.); 2 Department of Oncology, Cancer Research UK and Medical Research Council Oxford Institute for Radiation Oncology, University of Oxford, Old Road Campus Research Building, Roosevelt Drive, Oxford OX3 7DQ, UK

**Keywords:** bioprosthetic heart valve, calcification, interleukin-6, genetic association, predictive model

## Abstract

Severe bioprosthetic mitral valve calcification is a significant problem in cardiovascular surgery. Unfortunately, clinical markers did not demonstrate efficacy in prediction of severe bioprosthetic mitral valve calcification. Here, we examined whether a genomics-based approach is efficient in predicting the risk of severe bioprosthetic mitral valve calcification. A total of 124 consecutive Russian patients who underwent mitral valve replacement surgery were recruited. We investigated the associations of the inherited variation in innate immunity, lipid metabolism and calcium metabolism genes with severe bioprosthetic mitral valve calcification. Genotyping was conducted utilizing the TaqMan assay. Eight gene polymorphisms were significantly associated with severe bioprosthetic mitral valve calcification and were therefore included into stepwise logistic regression which identified male gender, the T/T genotype of the rs3775073 polymorphism within the *TLR6* gene, the C/T genotype of the rs2229238 polymorphism within the *IL6R* gene, and the A/A genotype of the rs10455872 polymorphism within the *LPA* gene as independent predictors of severe bioprosthetic mitral valve calcification. The developed genomics-based model had fair predictive value with area under the receiver operating characteristic (ROC) curve of 0.73. In conclusion, our genomics-based approach is efficient for the prediction of severe bioprosthetic mitral valve calcification.

## 1. Introduction

Mitral valve calcification, accompanied by inflammation and lipid deposition, is associated with common cardiovascular risk factors and represents an important risk factor of mitral valve disease [1,2]. Currently, there is no efficient approach for the prevention of mitral valve disease progression, with valve replacement being the only treatment option [1]. However, bioprosthetic mitral valves also frequently undergo severe calcification which is able to cause bioprosthetic valve failure and may require repeated valve replacement surgery, a major clinical intervention [1]. Even the widely established Carpentier-Edwards Perimount and Medtronic Mosaic bioprosthetic mitral valves undergo severe calcification in up to 20% of patients <60 years [3,4].

Unfortunately, there is still no clinical model for the prediction of severe bioprosthetic mitral valve calcification. A previous study by our research group did not reveal any significant clinical predictors of this condition [5]. Mitral valve calcification is frequent among family members [6] but genomic markers of native and bioprosthetic mitral valve calcification are still almost unknown [7]. Nevertheless, their identification may assist in revealing the underlying mechanisms of these conditions. This, in turn, may improve treatment of mitral valve disease.

Progress in genotyping technologies resulted in many studies on the association of single nucleotide polymorphisms (SNPs) with human diseases [8]. SNPs can lead to a number of consequences depending on their location in the genome [9]. As known, SNPs within the noncoding regions are able to affect mRNA splicing or even transcription initiation, while SNPs within the coding regions may alter protein folding, stability, and expression, or influence posttranslational modifications [9]. Here, we investigated whether SNPs within innate immunity, lipid metabolism and calcium metabolism genes are significant predictors of severe bioprosthetic mitral valve calcification.

## 2. Results

We identified eight SNPs being significantly associated with severe bioprosthetic mitral valve calcification (Table 1).

The C allele of the rs1800796 polymorphism within the *TLR6* gene, the T allele of the rs1205 polymorphism within the *CRP* gene, and the G allele of the rs10455872 polymorphism within the *LPA* gene were associated with decreased risk of severe bioprosthetic mitral valve calcification. In contrast, the A allele of the rs5743810 polymorphism within the *TLR6* gene, the C/T genotype of the rs2229238 polymorphism within the *IL6R* gene, the A/G genotype of the rs1800871 polymorphism and the T/G genotype of the rs1800872 polymorphism within the *IL10* gene, and the G/G genotype of the rs13290979 polymorphism within the *NOTCH1* gene were associated with increased risk of severe bioprosthetic mitral valve calcification. To perform an additional quality control step, we tested six non-relevant SNPs within the genes encoding coagulation factors and integrin beta 3, a protein responsible for platelet aggregation. Expectedly, we did not find any significant associations with severe bioprosthetic mitral valve calcification.

We then carried out a stepwise logistic regression to reveal independent predictive markers of severe bioprosthetic mitral valve calcification. Out of eight markers revealed by genetic association analysis, only three remained significant (Table 2).

A final model for prediction of severe bioprosthetic mitral valve calcification included male gender, the T/T genotype of the rs3775073 polymorphism within the *TLR6* gene, the C/T genotype of the rs2229238 polymorphism within the *IL6R* gene, and the A/A genotype of the rs10455872 polymorphism within the *LPA* gene. The area under the ROC curve of 0.73 demonstrated the fair predictive value of the model.

## 3. Discussion

Previous studies vaguely uncovered the genetic susceptibility to mitral annular calcification. Novaro et al. [10] and Tangri et al. [11] did not detect significant associations between polymorphisms within *apoE* (gene encoding apolipoprotein E), *Klotho*, *β-Klotho*, and *FGF-23* (genes encoding proteins constituting one of the calcium phosphate homeostasis pathways) genes and mitral annular calcification. Davutoglu and Nacak [12] reported that the I allele of the rs4340 polymorphism within the *ACE* gene (encoding angiotensin-converting enzyme) correlated with a higher risk of mitral annular calcification. Moreover, a study by Thanassoulis et al. [13] revealed two *IL1F9* (gene encoding IL-36γ/IL-1F9 protein) gene polymorphisms, rs17659543 and rs13415097, being significantly associated with higher risk of mitral annular calcification.

However, there are no published data on genetic susceptibility to bioprosthetic mitral valve calcification. In addition, there is no any model for the prediction of bioprosthetic mitral valve calcification. Here we identified the T/T genotype of the rs3775073 polymorphism within the *TLR6* gene, the C/T genotype of the rs2229238 polymorphism within the *IL6R* gene, and the A/A genotype of the rs10455872 polymorphism within the *LPA* gene as the independent predictive markers of severe bioprosthetic mitral valve calcification. Moreover, we developed a predictive model with the fair discriminative power. Nevertheless, area under the receiver operating characteristic (ROC) curve of 0.73 indicates a number of other relevant predictive markers to be discovered.

A previous study by our research group found that the C/T genotype of the rs2229238 polymorphism within the *IL6R* gene is significantly associated with a higher IL-6 plasma level compared to the C/C and T/T genotypes [14]. It is worth noting that IL-6 is associated with heart valve calcification in general and with mitral annular calcification in particular [15,16]. Therefore, we hypothesize that the C/T genotype of the rs2229238 polymorphism within the *IL6R* gene may increase IL-6 plasma level and may thus promote bioprosthetic mitral valve calcification.

Our study had a considerable shortcoming: we recruited a relatively small sample due to a limited number of mitral valve replacements. However, we tested six irrelevant SNPs for the occasional associations, expectedly with a negative result. This approach was used to increase statistical confidence when using a small sample size.

Our findings may have clinical applications. A genomics-based model for the prediction of severe bioprosthetic mitral valve calcification can be used in choosing between mechanical and bioprosthetic mitral valves for mitral valve replacement surgery. For carriers of the high risk genotypes, mechanical heart valves which are resistant to calcification may be an appropriate option (reviewed by Bre et al. [17]). Further investigations on larger samples are necessary to confirm our results.

## 4. Materials and Methods

### 4.1. Population

Inclusion criteria were: (1) living in Kemerovo Region for ≥2 generations; (2) Russian ethnicity; (3) mitral valve replacement surgery due to mitral valve disease; and (4) written informed consent. Exclusion criteria were: (1) belonging to the immigrant or aboriginal populations; (2) previous cancer diagnosis; (3) concomitant mental disorders and/or autoimmune diseases; and (4) refusal to sign a written informed consent.

We recruited 140 patients admitted to our Research Institute who underwent mitral valve replacement surgery due to mitral valve disease in 2006–2007. After exclusion of 16 patients due to the above-mentioned criteria, the study group finally included 124 patients (Table 3).

Half of them (*n* = 62) had severe bioprosthetic mitral valve calcification within 8 years post-implantation and therefore represented a case group; remaining subjects (*n* = 62) without severe bioprosthetic mitral valve calcification were considered as the controls (Table 4). The local ethical committee approved the study protocol. All the participants provided written informed consent after the study was fully explained.

**Figure 1:**
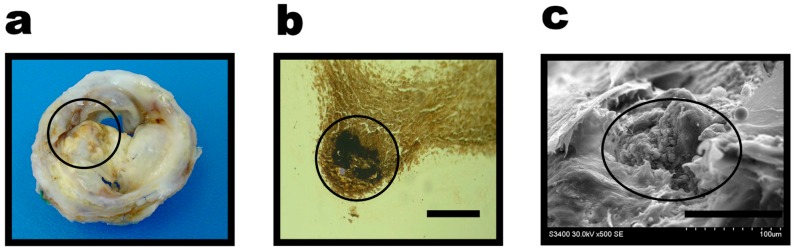
Bioprosthetic valve calcification: (**a**) explanted bioprosthetic heart valve; (**b**) von Kossa staining, scale bar = 50 µm; (**c**) scanning electron microscopy. Calcified areas are indicated as black circles.

The diagnosis of mitral valve disease and decision on mitral valve replacement surgery were performed in accordance with the respective American guidelines [18]. For the mitral valve replacement, we used KemCor and PeriCor bioprosthetic valves (NeoCor, Russian Federation) crosslinked with ethylene glycol diglycidyl ether for conferring resistance to oxidation and enzymatic degradation [19]. Functional conditions of the bioprosthetic valves were annually assessed by echocardiography. After the explantation of failing bioprosthesis (Figure 1a), bioprosthetic mitral valve calcification was verified by von Kossa staining (Figure 1b) and scanning electron microscopy (Figure 1c).

**Figure 2:**
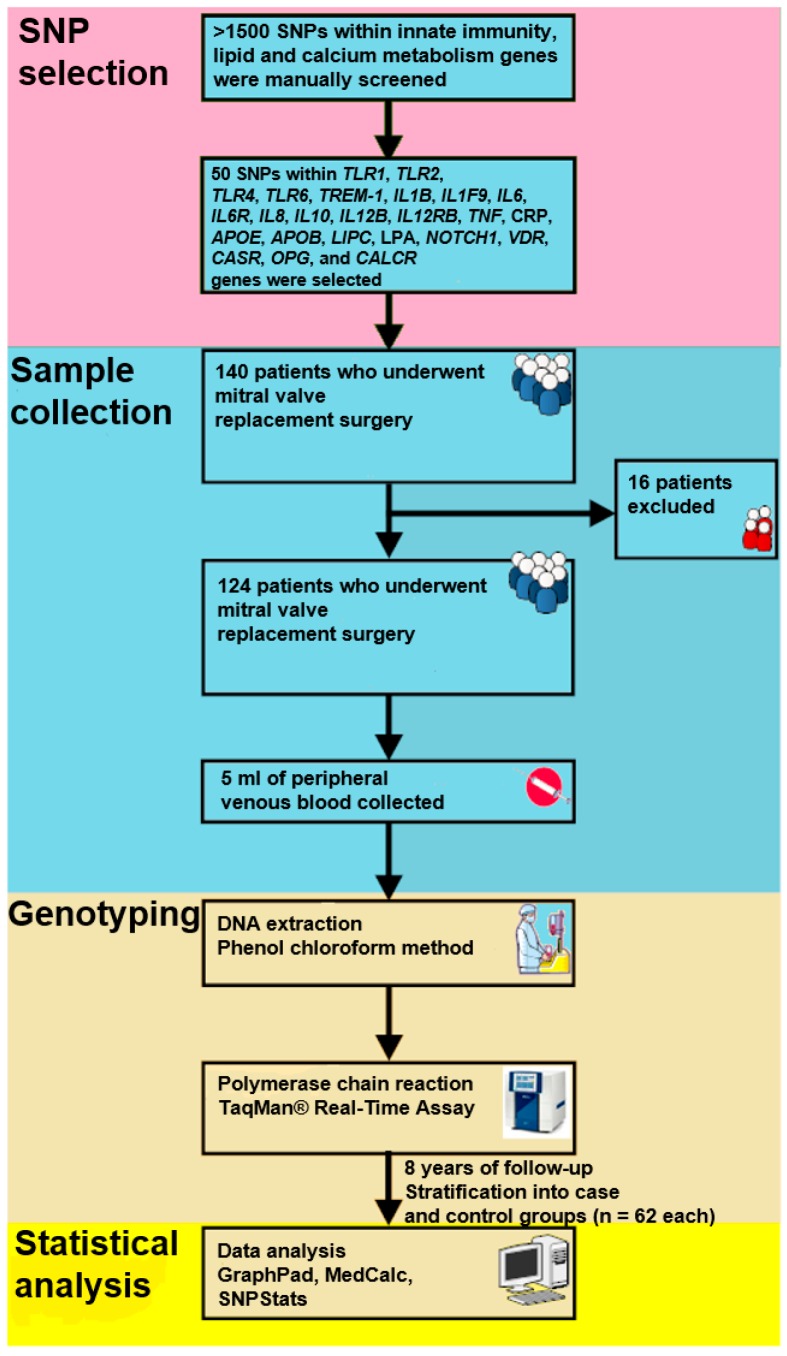
Study workflow.

The study workflow is shown in the Figure 2.

### 4.2. SNP Selection and Genotyping

For this study, we defined four main criteria for SNP selection: (1) location within innate immunity, lipid metabolism, or calcium metabolism genes; (2) minor allele frequency ≥5% for Russian population tested with HapMap; (3) functional consequences; and (4) few or no studies on the role of the SNP in mitral valve calcification. The National Center for Biotechnology Information dbSNP, SNPinfo, and SNPnexus databases were utilized for the SNP selection [20,21]. In total, we selected 50 SNPs within 24 genes (Table 5).

The procedures of DNA extraction and genotyping were the same as previously described [22,23,24]. Table 5 demonstrates the sequence-specific primers for genotyped SNPs. Laboratory staff was blinded to patient status, and one-tenth of the samples was repeatedly genotyped for quality control.

### 4.3. Statistical Analysis

The statistical analysis was performed as in [22,23,24] using the SNPStats software [25]. To further define independent predictors of severe bioprosthetic mitral valve calcification, we carried out stepwise logistic regression with the plotting of the ROC curve and area under the curve.

## Figures and Tables

**Table 1:** Association of the polymorphisms within innate immunity genes, genes of lipid metabolism, and genes of calcium metabolism with severe bioprosthetic mitral valve calcification.

Model	Genotype	Without Severe Bioprosthetic Mitral Valve Calcification	With Severe Bioprosthetic Mitral Valve Calcification	OR (95% CI)	*p*-Value	AIC	HWE
*TLR1* rs5743551							
Codominant	T/T	31 (50%)	35 (56.5%)	1.00	0.39	171.7	0.06
	C/T	30 (48.4%)	25 (40.3%)	0.69 (0.32–1.45)			
	C/C	1 (1.6%)	2 (3.2%)	2.71 (0.22–33.36)			
Dominant	T/T	31 (50%)	35 (56.5%)	1.00	0.42	170.9	
	C/T-C/C	31 (50%)	27 (43.5%)	0.74 (0.36–1.54)			
Recessive	T/T-C/T	61 (98.4%)	60 (96.8%)	1.00	0.35	170.7	
	C/C	1 (1.6%)	2 (3.2%)	3.17 (0.26–38.23)			
Overdominant	T/T-C/C	32 (51.6%)	37 (59.7%)	1.00	0.27	170.4	
	C/T	30 (48.4%)	25 (40.3%)	0.66 (0.31–1.39)			
Log-additive	---	---	---	0.85 (0.44–1.67)	0.64	171.4	
*TLR1* rs5743611							
Codominant	C/C	38 (61.3%)	36 (58.1%)	1.00	0.76	173	0.61
	C/G	21 (33.9%)	21 (33.9%)	1.00 (0.46–2.19)			
	G/G	3 (4.8%)	5 (8.1%)	1.74 (0.38–7.96)			
Dominant	C/C	38 (61.3%)	36 (58.1%)	1.00	0.81	171.5	
	C/G-G/G	24 (38.7%)	26 (41.9%)	1.10 (0.52–2.30)			
Recessive	C/C-C/G	59 (95.2%)	57 (91.9%)	1.00	0.46	171	
	G/G	3 (4.8%)	5 (8.1%)	1.74 (0.39–7.75)			
Overdominant	C/C-G/G	41 (66.1%)	41 (66.1%)	1.00	0.89	171.6	
	C/G	21 (33.9%)	21 (33.9%)	0.94 (0.44–2.04)			
Log-additive	---	---	---	1.16 (0.64–2.09)	0.62	171.3	
*TLR2* rs5743708							
---	G/G	57 (91.9%)	56 (90.3%)	1.00	0.67	171.4	0.99
	A/G	5 (8.1%)	6 (9.7%)	1.33 (0.36–4.92)			
*TLR2* rs3804099							
Codominant	T/T	23 (37.1%)	18 (29%)	1.00	0.37	171.6	0.06
	C/T	33 (53.2%)	37 (59.7%)	1.80 (0.79–4.13)			
	C/C	6 (9.7%)	7 (11.3%)	1.35 (0.36–5.06)			
Dominant	T/T	23 (37.1%)	18 (29%)	1.00	0.18	169.8	
	C/T-C/C	39 (62.9%)	44 (71%)	1.72 (0.77–3.82)			
Recessive	T/T-C/T	56 (90.3%)	55 (88.7%)	1.00	0.93	171.6	
	C/C	6 (9.7%)	7 (11.3%)	0.94 (0.28–3.17)			
Overdominant	T/T-C/C	29 (46.8%)	25 (40.3%)	1.00	0.18	169.8	
	C/T	33 (53.2%)	37 (59.7%)	1.68 (0.78–3.60)			
Log-additive	---	---	---	1.34 (0.73–2.46)	0.33	170.6	
*TLR4* rs4986790							
Codominant	A/A	53 (85.5%)	53 (85.5%)	1.00	0.46	172	0.53
	A/G	8 (12.9%)	9 (14.5%)	1.19 (0.41–3.45)			
	G/G	1 (1.6%)	0 (0%)	0.00 (0.00–0.00)			
Dominant	A/A	53 (85.5%)	53 (85.5%)	1.00	0.95	171.6	
	A/G-G/G	9 (14.5%)	9 (14.5%)	1.03 (0.37–2.91)			
Recessive	A/A-A/G	61 (98.4%)	62 (100%)	1.00	0.23	170.1	
	G/G	1 (1.6%)	0 (0%)	0.00 (0.00–0.00)			
Overdominant	A/A-G/G	54 (87.1%)	53 (85.5%)	1.00	0.73	171.5	
	A/G	8 (12.9%)	9 (14.5%)	1.20 (0.41–3.50)			
Log-additive	---	---	---	0.91 (0.35–2.35)	0.85	171.5	
*TLR4* rs4986791							
Codominant	C/C	53 (85.5%)	53 (85.5%)	1.00	0.98	173.5	0.17
	C/T	8 (12.9%)	8 (12.9%)	1.00 (0.33–2.97)			
	T/T	1 (1.6%)	1 (1.6%)	1.36 (0.08–23.62)			
Dominant	C/C	53 (85.5%)	53 (85.5%)	1.00	0.95	171.6	
	C/T-T/T	9 (14.5%)	9 (14.5%)	1.03 (0.37–2.91)			
Recessive	C/C-C/T	61 (98.4%)	61 (98.4%)	1.00	0.83	171.5	
	T/T	1 (1.6%)	1 (1.6%)	1.36 (0.08–23.58)			
Overdominant	C/C-T/T	54 (87.1%)	54 (87.1%)	1.00	0.99	171.6	
	C/T	8 (12.9%)	8 (12.9%)	0.99 (0.33–2.95)			
Log-additive	---	---	---	1.05 (0.43–2.56)	0.91	171.6	
*TLR6* rs3775073							
Codominant	T/T	12 (19.4%)	20 (32.3%)	1.00	0.092	168.8	0.72
	T/C	32 (51.6%)	33 (53.2%)	0.71 (0.29–1.75)			
	C/C	18 (29%)	9 (14.5%)	0.31 (0.10–0.94)			
Dominant	T/T	12 (19.4%)	20 (32.3%)	1.00	0.18	169.8	
	T/C-C/C	50 (80.7%)	42 (67.7%)	0.56 (0.24–1.32)			
Recessive	T/T-T/C	44 (71%)	53 (85.5%)	1.00	0.04	167.4	
	C/C	18 (29%)	9 (14.5%)	0.39 (0.15–0.98)			
Overdominant	T/T-C/C	30 (48.4%)	29 (46.8%)	1.00	0.59	171.3	
	T/C	32 (51.6%)	33 (53.2%)	1.22 (0.58–2.55)			
Log-additive	---	---	---	0.56 (0.32–0.98)	0.037	167.2	
*TLR6* rs5743810							
Codominant	G/G	35 (56.5%)	24 (38.7%)	1.00	0.09	168.8	0.67
	A/G	25 (40.3%)	30 (48.4%)	1.57 (0.73–3.38)			
	A/A	2 (3.2%)	8 (12.9%)	5.19 (0.97–27.93)			
Dominant	G/G	35 (56.5%)	24 (38.7%)	1.00	0.11	169	
	A/G-A/A	27 (43.5%)	38 (61.3%)	1.83 (0.87–3.84)			
Recessive	G/G-A/G	60 (96.8%)	54 (87.1%)	1.00	0.062	168.1	
	A/A	2 (3.2%)	8 (12.9%)	4.17 (0.81–21.53)			
Overdominant	G/G-A/A	37 (59.7%)	32 (51.6%)	1.00	0.53	171.2	
	A/G	25 (40.3%)	30 (48.4%)	1.26 (0.61–2.64)			
Log-additive	---	---	---	1.87 (1.02–3.44)	0.039	167.3	
*TREM1* rs2234246							
Codominant	C/C	15 (24.2%)	18 (29%)	1.00	0.54	172.3	0.99
	C/T	29 (46.8%)	33 (53.2%)	1.05 (0.43–2.52)			
	T/T	18 (29%)	11 (17.7%)	0.63 (0.22–1.81)			
Dominant	C/C	15 (24.2%)	18 (29%)	1.00	0.8	171.5	
	C/T-T/T	47 (75.8%)	44 (71%)	0.90 (0.39–2.07)			
Recessive	C/C-C/T	44 (71%)	51 (82.3%)	1.00	0.27	170.3	
	T/T	18 (29%)	11 (17.7%)	0.61 (0.25–1.47)			
Overdominant	C/C-T/T	33 (53.2%)	29 (46.8%)	1.00	0.48	171.1	
	C/T	29 (46.8%)	33 (53.2%)	1.30 (0.63–2.70)			
Log-additive	---	---	---	0.80 (0.47–1.36)	0.41	170.9	
*TREM1* rs4711668							
Codominant	C/C	26 (41.9%)	21 (33.9%)	1.00	0.46	172	0.85
	T/C	30 (48.4%)	30 (48.4%)	1.29 (0.58–2.85)			
	T/T	6 (9.7%)	11 (17.7%)	2.07 (0.64–6.75)			
Dominant	C/C	26 (41.9%)	21 (33.9%)	1.00	0.35	170.7	
	T/C-T/T	36 (58.1%)	41 (66.1%)	1.43 (0.67–3.04)			
Recessive	C/C-T/C	56 (90.3%)	51 (82.3%)	1.00	0.29	170.4	
	T/T	6 (9.7%)	11 (17.7%)	1.80 (0.60–5.37)			
Overdominant	C/C-T/T	32 (51.6%)	32 (51.6%)	1.00	0.87	171.5	
	T/C	30 (48.4%)	30 (48.4%)	1.07 (0.51–2.21)			
Log-additive	---	---	---	1.40 (0.81–2.41)	0.23	170.1	
*TREM1* rs3804277							
Codominant	C/C	16 (25.8%)	18 (29%)	1.00	0.52	172.3	0.86
	C/T	28 (45.2%)	33 (53.2%)	1.13 (0.47–2.69)			
	T/T	18 (29%)	11 (17.7%)	0.66 (0.23–1.89)			
Dominant	C/C	16 (25.8%)	18 (29%)	1.00	0.92	171.6	
	C/T-T/T	46 (74.2%)	44 (71%)	0.96 (0.42–2.18)			
Recessive	C/C-C/T	44 (71%)	51 (82.3%)	1.00	0.27	170.3	
	T/T	18 (29%)	11 (17.7%)	0.61 (0.25–1.47)			
Overdominant	C/C-T/T	34 (54.8%)	29 (46.8%)	1.00	0.41	170.9	
	C/T	28 (45.2%)	33 (53.2%)	1.36 (0.66–2.83)			
Log-additive	---	---	---	0.82 (0.49–1.39)	0.47	171	
*TREM1* rs2234237							
Codominant	T/T	49 (79%)	50 (80.7%)	1.00	0.39	171.7	0.99
	A/T	13 (21%)	11 (17.7%)	0.69 (0.27–1.79)			
	A/A	0 (0%)	1 (1.6%)	0.00 (0.00–0.00)			
Dominant	T/T	49 (79%)	50 (80.7%)	1.00	0.55	171.2	
	A/T-A/A	13 (21%)	12 (19.4%)	0.76 (0.30–1.92)			
Recessive	T/T-A/T	62 (100%)	61 (98.4%)	1.00	0.26	170.3	
	A/A	0 (0%)	1 (1.6%)	0.00 (0.00–0.00)			
Overdominant	T/T-A/A	49 (79%)	51 (82.3%)	1.00	0.41	170.9	
	A/T	13 (21%)	11 (17.7%)	0.67 (0.26–1.74)			
Log-additive	---	---	---	0.86 (0.36–2.05)	0.73	171.5	
*TREM1* rs6910730							
Codominant	A/A	49 (79%)	48 (77.4%)	1.00	0.49	172.2	0.99
	A/G	13 (21%)	13 (21%)	0.84 (0.34–2.10)			
	G/G	0 (0%)	1 (1.6%)	0.00 (0.00–0.00))			
Dominant	A/A	49 (79%)	48 (77.4%)	1.00	0.83	171.5	
	A/G-G/G	13 (21%)	14 (22.6%)	0.91 (0.37–2.24)			
Recessive	A/A-A/G	62 (100%)	61 (98.4%)	1.00	0.26	170.3	
	G/G	0 (0%)	1 (1.6%)	0.00 (0.00–0.00)			
Overdominant	A/A-G/G	49 (79%)	49 (79%)	1.00	0.67	171.4	
	A/G	13 (21%)	13 (21%)	0.82 (0.33–2.05)			
Log-additive	---	---	---	1.00 (0.43–2.34)	1	171.6	
*TREM1* rs1817537							
Codominant	C/C	16 (25.8%)	18 (29%)	1.00	0.52	172.3	0.86
	C/G	28 (45.2%)	33 (53.2%)	1.13 (0.47–2.69)			
	G/G	18 (29%)	11 (17.7%)	0.66 (0.23–1.89)			
Dominant	C/C	16 (25.8%)	18 (29%)	1.00	0.92	171.6	
	C/G-G/G	46 (74.2%)	44 (71%)	0.96 (0.42–2.18)			
Recessive	C/C-C/G	44 (71%)	51 (82.3%)	1.00	0.27	170.3	
	G/G	18 (29%)	11 (17.7%)	0.61 (0.25–1.47)			
Overdominant	C/C-G/G	34 (54.8%)	29 (46.8%)	1.00	0.41	170.9	
	C/G	28 (45.2%)	33 (53.2%)	1.36 (0.66–2.83)			
Log-additive	---	---	---	0.82 (0.49–1.39)	0.47	171	
*TREM1* rs9471535							
Codominant	T/T	49 (79%)	50 (80.7%)	1.00	0.39	171.7	0.99
	C/T	13 (21%)	11 (17.7%)	0.69 (0.27–1.79)			
	C/C	0 (0%)	1 (1.6%)	0.00 (0.00–0.00)			
Dominant	T/T	49 (79%)	50 (80.7%)	1.00	0.55	171.2	
	C/T-C/C	13 (21%)	12 (19.4%)	0.76 (0.30–1.92)			
Recessive	T/T-C/T	62 (100%)	61 (98.4%)	1.00	0.26	170.3	
	C/C	0 (0%)	1 (1.6%)	0.00 (0.00–0.00)			
Overdominant	T/T-C/C	49 (79%)	51 (82.3%)	1.00	0.41	170.9	
	C/T	13 (21%)	11 (17.7%)	0.67 (0.26–1.74)			
Log-additive	---	---	---	0.86 (0.36–2.05)	0.73	171.5	
*TREM1* rs7768162							
Codominant	G/G	26 (41.9%)	21 (33.9%)	1.00	0.57	172.4	0.25
	A/G	31 (50%)	33 (53.2%)	1.35 (0.62–2.96)			
	A/A	5 (8.1%)	8 (12.9%)	1.88 (0.51–6.85)			
Dominant	G/G	26 (41.9%)	21 (33.9%)	1.00	0.35	170.7	
	A/G-A/A	36 (58.1%)	41 (66.1%)	1.43 (0.67–3.04)			
Recessive	G/G-A/G	57 (91.9%)	54 (87.1%)	1.00	0.46	171	
	A/A	5 (8.1%)	8 (12.9%)	1.58 (0.47–5.29)			
Overdominant	G/G-A/A	31 (50%)	29 (46.8%)	1.00	0.66	171.4	
	A/G	31 (50%)	33 (53.2%)	1.18 (0.57–2.45)			
Log-additive	---	---	---	1.36 (0.77–2.43)	0.29	170.4	
*IL1B* rs16944							
Codominant	G/G	26 (41.9%)	25 (40.3%)	1.00	0.88	173.3	0.42
	G/A	31 (50%)	30 (48.4%)	0.92 (0.42–1.99)			
	A/A	5 (8.1%)	7 (11.3%)	1.27 (0.33–4.79)			
Dominant	G/G	26 (41.9%)	25 (40.3%)	1.00	0.92	171.6	
	G/A-A/A	36 (58.1%)	37 (59.7%)	0.96 (0.46–2.04)			
Recessive	G/G-G/A	57 (91.9%)	55 (88.7%)	1.00	0.65	171.4	
	A/A	5 (8.1%)	7 (11.3%)	1.33 (0.38–4.66)			
Overdominant	G/G-A/A	31 (50%)	32 (51.6%)	1.00	0.72	171.4	
	G/A	31 (50%)	30 (48.4%)	0.88 (0.42–1.82)			
Log-additive	---	---	---	1.04 (0.58–1.86)	0.89	171.6	
*IL1B* rs1143634							
Codominant	G/G	30 (48.4%)	40 (64.5%)	1.00	0.18	170.2	0.48
	G/A	27 (43.5%)	17 (27.4%)	0.48 (0.21–1.06)			
	A/A	5 (8.1%)	5 (8.1%)	0.63 (0.16–2.54)			
Dominant	G/G	30 (48.4%)	40 (64.5%)	1.00	0.07	168.3	
	G/A-A/A	32 (51.6%)	22 (35.5%)	0.50 (0.24–1.07)			
Recessive	G/G-G/A	57 (91.9%)	57 (91.9%)	1.00	0.79	171.5	
	A/A	5 (8.1%)	5 (8.1%)	0.83 (0.21–3.24)			
Overdominant	G/G-A/A	35 (56.5%)	45 (72.6%)	1.00	0.084	168.6	
	G/A	27 (43.5%)	17 (27.4%)	0.51 (0.23–1.10)			
Log-additive	---	---	---	0.64 (0.36–1.15)	0.13	169.3	
*IL1F9* rs17659543							
Codominant	C/C	49 (79%)	48 (78.7%)	1.00	0.65	172	0.99
	C/T	12 (19.4%)	13 (21.3%)	1.03 (0.41–2.55)			
	T/T	1 (1.6%)	0 (0%)	0.00 (0.00–0.00)			
Dominant	C/C	49 (79%)	48 (78.7%)	1.00	0.94	170.8	
	C/T-T/T	13 (21%)	13 (21.3%)	0.97 (0.40–2.37)			
Recessive	C/C-C/T	61 (98.4%)	61 (100%)	1.00	0.35	170	
	T/T	1 (1.6%)	0 (0%)	0.00 (0.00–0.00)			
Overdominant	C/C-T/T	50 (80.7%)	48 (78.7%)	1.00	0.93	170.8	
	C/T	12 (19.4%)	13 (21.3%)	1.04 (0.42–2.59)			
Log-additive	---	---	---	0.91 (0.39–2.13)	0.83	170.8	
*IL6* rs1554606							
Codominant	T/T	17 (27.4%)	13 (21%)	1.00	0.59	172.5	0.47
	G/T	30 (48.4%)	37 (59.7%)	1.43 (0.58–3.52)			
	G/G	15 (24.2%)	12 (19.4%)	0.94 (0.32–2.82)			
Dominant	T/T	17 (27.4%)	13 (21%)	1.00	0.58	171.3	
	G/T-G/G	45 (72.6%)	49 (79%)	1.27 (0.54–3.02)			
Recessive	T/T-G/T	47 (75.8%)	50 (80.7%)	1.00	0.5	171.1	
	G/G	15 (24.2%)	12 (19.4%)	0.73 (0.30–1.80)			
Overdominant	T/T-G/G	32 (51.6%)	25 (40.3%)	1.00	0.3	170.5	
	G/T	30 (48.4%)	37 (59.7%)	1.47 (0.70–3.07)			
Log-additive	---	---	---	0.98 (0.57–1.69)	0.94	171.6	
*IL6* rs1800796							
Codominant	G/G	53 (85.5%)	49 (79%)	1.00	0.69	172.8	0.10
	C/G	8 (12.9%)	11 (17.7%)	1.51 (0.54–4.24)			
	C/C	1 (1.6%)	2 (3.2%)	1.66 (0.14–20.27)			
Dominant	G/G	53 (85.5%)	49 (79%)	1.00	0.39	170.8	
	C/G-C/C	9 (14.5%)	13 (21%)	1.53 (0.58–4.06)			
Recessive	G/G-C/G	61 (98.4%)	60 (96.8%)	1.00	0.73	171.5	
	C/C	1 (1.6%)	2 (3.2%)	1.54 (0.13–18.69)			
Overdominant	G/G-C/C	54 (87.1%)	51 (82.3%)	1.00	0.44	171	
	C/G	8 (12.9%)	11 (17.7%)	1.49 (0.53–4.17)			
Log-additive	---	---	---	1.42 (0.62–3.26)	0.4	170.9	
*IL6* rs2069827							
Codominant	G/G	48 (77.4%)	51 (82.3%)	1.00	0.68	172.8	0.63
	G/T	13 (21%)	10 (16.1%)	0.66 (0.26–1.69)			
	T/T	1 (1.6%)	1 (1.6%)	0.87 (0.05–15.09)			
Dominant	G/G	48 (77.4%)	51 (82.3%)	1.00	0.39	170.8	
	G/T-T/T	14 (22.6%)	11 (17.7%)	0.67 (0.27–1.68)			
Recessive	G/G-G/T	61 (98.4%)	61 (98.4%)	1.00	0.97	171.6	
	T/T	1 (1.6%)	1 (1.6%)	0.95 (0.06–16.33)			
Overdominant	G/G-T/T	49 (79%)	52 (83.9%)	1.00	0.39	170.8	
	G/T	13 (21%)	10 (16.1%)	0.66 (0.26–1.69)			
Log-additive	---	---	---	0.73 (0.32–1.64)	0.44	171	
*IL6R* rs2228145							
Codominant	A/A	25 (40.3%)	28 (45.2%)	1.00	0.81	173.2	0.99
	C/A	29 (46.8%)	28 (45.2%)	1.00 (0.46–2.18)			
	C/C	8 (12.9%)	6 (9.7%)	0.68 (0.20–2.34)			
Dominant	A/A	25 (40.3%)	28 (45.2%)	1.00	0.84	171.5	
	C/A-C/C	37 (59.7%)	34 (54.8%)	0.93 (0.44–1.94)			
Recessive	A/A-C/A	54 (87.1%)	56 (90.3%)	1.00	0.52	171.2	
	C/C	8 (12.9%)	6 (9.7%)	0.68 (0.21–2.20)			
Overdominant	A/A-C/C	33 (53.2%)	34 (54.8%)	1.00	0.83	171.5	
	C/A	29 (46.8%)	28 (45.2%)	1.08 (0.52–2.27)			
Log-additive	---	---	---	0.88 (0.51–1.53)	0.65	171.4	
*IL6R* rs2229238							
Codominant	C/C	42 (67.7%)	35 (56.5%)	1.00	0.03	166.6	0.30
	C/T	14 (22.6%)	25 (40.3%)	2.48 (1.07–5.73)			
	T/T	6 (9.7%)	2 (3.2%)	0.40 (0.07–2.25)			
Dominant	C/C	42 (67.7%)	35 (56.5%)	1.00	0.12	169.2	
	C/T-T/T	20 (32.3%)	27 (43.5%)	1.83 (0.84–3.96)			
Recessive	C/C-C/T	56 (90.3%)	60 (96.8%)	1.00	0.13	169.3	
	T/T	6 (9.7%)	2 (3.2%)	0.30 (0.05–1.59)			
Overdominant	C/C-T/T	48 (77.4%)	37 (59.7%)	1.00	0.016	165.8	
	C/T	14 (22.6%)	25 (40.3%)	2.70 (1.18–6.16)			
Log-additive	---	---	---	1.21 (0.66–2.21)	0.54	171.2	
*IL8* rs2227306							
Codominant	C/C	20 (32.3%)	20 (32.3%)	1.00	0.97	173.5	0.72
	C/T	29 (46.8%)	30 (48.4%)	1.05 (0.45–2.43)			
	T/T	13 (21%)	12 (19.4%)	0.92 (0.33–2.61)			
Dominant	C/C	20 (32.3%)	20 (32.3%)	1.00	0.99	171.6	
	C/T-T/T	42 (67.7%)	42 (67.7%)	1.01 (0.46–2.22)			
Recessive	C/C-C/T	49 (79%)	50 (80.7%)	1.00	0.82	171.5	
	T/T	13 (21%)	12 (19.4%)	0.90 (0.36–2.23)			
Overdominant	C/C-T/T	33 (53.2%)	32 (51.6%)	1.00	0.84	171.5	
	C/T	29 (46.8%)	30 (48.4%)	1.08 (0.52–2.26)			
Log-additive	---	---	---	0.97 (0.58–1.62)	0.9	171.6	
*IL10* rs1800871							
Codominant	G/G	34 (54.8%)	31 (50%)	1.00	0.029	166.5	0.09
	A/G	24 (38.7%)	31 (50%)	1.81 (0.83–3.95)			
	A/A	4 (6.5%)	0 (0%)	0.00 (0.00–0.00)			
Dominant	G/G	34 (54.8%)	31 (50%)	1.00	0.26	170.3	
	A/G-A/A	28 (45.2%)	31 (50%)	1.55 (0.72–3.32)			
Recessive	G/G-A/G	58 (93.5%)	62 (100%)	1.00	0.029	166.8	
	A/A	4 (6.5%)	0 (0%)	0.00 (0.00–0.00)			
Overdominant	G/G-A/A	38 (61.3%)	31 (50%)	1.00	0.07	168.3	
	A/G	24 (38.7%)	31 (50%)	2.02 (0.93–4.38)			
Log-additive	---	---	---	1.15 (0.59–2.24)	0.68	171.4	
*IL10* rs1800872							
Codominant	G/G	34 (54.8%)	30 (49.2%)	1.00	0.028	165.7	0.09
	T/G	24 (38.7%)	31 (50.8%)	1.84 (0.84–4.00)			
	T/T	4 (6.5%)	0 (0%)	0.00 (0.00–0.00)			
Dominant	G/G	34 (54.8%)	30 (49.2%)	1.00	0.24	169.5	
	T/G-T/T	28 (45.2%)	31 (50.8%)	1.57 (0.73–3.36)			
Recessive	G/G-T/G	58 (93.5%)	61 (100%)	1.00	0.029	166.1	
	T/T	4 (6.5%)	0 (0%)	0.00 (0.00–0.00)			
Overdominant	G/G-T/T	38 (61.3%)	30 (49.2%)	1.00	0.065	167.4	
	T/G	24 (38.7%)	31 (50.8%)	2.05 (0.95–4.44)			
Log-additive	---	---	---	1.17 (0.60–2.27)	0.65	170.6	
*IL10* rs1800896							
Codominant	T/T	17 (27.4%)	16 (25.8%)	1.00	0.46	172	0.86
	T/C	30 (48.4%)	34 (54.8%)	1.41 (0.58–3.41)			
	C/C	15 (24.2%)	12 (19.4%)	0.79 (0.27–2.34)			
Dominant	T/T	17 (27.4%)	16 (25.8%)	1.00	0.68	171.4	
	T/C-C/C	45 (72.6%)	46 (74.2%)	1.19 (0.52–2.74)			
Recessive	T/T-T/C	47 (75.8%)	50 (80.7%)	1.00	0.33	170.6	
	C/C	15 (24.2%)	12 (19.4%)	0.64 (0.26–1.58)			
Overdominant	T/T-C/C	32 (51.6%)	28 (45.2%)	1.00	0.24	170.2	
	T/C	30 (48.4%)	34 (54.8%)	1.56 (0.74–3.29)			
Log-additive	---	---	---	0.92 (0.54–1.56)	0.75	171.5	
*IL12B* rs3212227							
Codominant	T/T	38 (61.3%)	36 (58.1%)	1.00	0.77	173	0.80
	G/T	21 (33.9%)	22 (35.5%)	1.30 (0.59–2.85)			
	G/G	3 (4.8%)	4 (6.5%)	1.42 (0.28–7.32)			
Dominant	T/T	38 (61.3%)	36 (58.1%)	1.00	0.47	171.1	
	G/T-G/G	24 (38.7%)	26 (41.9%)	1.32 (0.62–2.79)			
Recessive	T/T-G/T	59 (95.2%)	58 (93.5%)	1.00	0.75	171.5	
	G/G	3 (4.8%)	4 (6.5%)	1.29 (0.26–6.47)			
Overdominant	T/T-G/G	41 (66.1%)	40 (64.5%)	1.00	0.55	171.2	
	G/T	21 (33.9%)	22 (35.5%)	1.26 (0.58–2.73)			
Log-additive	---	---	---	1.25 (0.67–2.31)	0.48	171.1	
*IL12RB* rs375947							
Codominant	A/A	27 (43.5%)	26 (41.9%)	1.00	0.77	173.1	0.84
	A/G	26 (41.9%)	29 (46.8%)	1.21 (0.56–2.66)			
	G/G	9 (14.5%)	7 (11.3%)	0.82 (0.25–2.67)			
Dominant	A/A	27 (43.5%)	26 (41.9%)	1.00	0.77	171.5	
	A/G-G/G	35 (56.5%)	36 (58.1%)	1.11 (0.53–2.33)			
Recessive	A/A-A/G	53 (85.5%)	55 (88.7%)	1.00	0.6	171.3	
	G/G	9 (14.5%)	7 (11.3%)	0.74 (0.25–2.26)			
Overdominant	A/A-G/G	36 (58.1%)	33 (53.2%)	1.00	0.52	171.2	
	A/G	26 (41.9%)	29 (46.8%)	1.27 (0.61–2.65)			
Log-additive	---	---	---	0.99 (0.58–1.69)	0.96	171.6	
*TNF* rs361525							
---	G/G	56 (90.3%)	60 (96.8%)	1.00	0.092	168.7	0.99
	A/G	6 (9.7%)	2 (3.2%)	0.25 (0.04–1.41)			
*TNF* rs1800629							
Codominant	G/G	48 (77.4%)	54 (87.1%)	1.00	0.39	171.7	0.06
	A/G	11 (17.7%)	7 (11.3%)	0.60 (0.21–1.73)			
	A/A	3 (4.8%)	1 (1.6%)	0.31 (0.03–3.19)			
Dominant	G/G	48 (77.4%)	54 (87.1%)	1.00	0.2	170	
	A/G-A/A	14 (22.6%)	8 (12.9%)	0.53 (0.20–1.42)			
Recessive	G/G-A/G	59 (95.2%)	61 (98.4%)	1.00	0.32	170.6	
	A/A	3 (4.8%)	1 (1.6%)	0.34 (0.03–3.42)			
Overdominant	G/G-A/A	51 (82.3%)	55 (88.7%)	1.00	0.38	170.8	
	A/G	11 (17.7%)	7 (11.3%)	0.62 (0.21–1.80)			
Log-additive	---	---	---	0.58 (0.26–1.29)	0.17	169.7	
*TNF* rs1799964							
Codominant	T/T	41 (66.1%)	41 (66.1%)	1.00	0.87	173.3	0.25
	C/T	17 (27.4%)	18 (29%)	0.95 (0.42–2.16)			
	C/C	4 (6.5%)	3 (4.8%)	0.65 (0.13–3.35)			
Dominant	T/T	41 (66.1%)	41 (66.1%)	1.00	0.78	171.5	
	C/T-C/C	21 (33.9%)	21 (33.9%)	0.90 (0.41–1.94)			
Recessive	T/T-C/T	58 (93.5%)	59 (95.2%)	1.00	0.61	171.3	
	C/C	4 (6.5%)	3 (4.8%)	0.66 (0.13–3.33)			
Overdominant	T/T-C/C	45 (72.6%)	44 (71%)	1.00	0.97	171.6	
	C/T	17 (27.4%)	18 (29%)	0.99 (0.44–2.21)			
Log-additive	---	---	---	0.88 (0.47–1.63)	0.68	171.4	
*CRP* rs3093077							
---	C/C	55 (88.7%)	56 (90.3%)	1.00	0.86	171.5	0.99
	A/C	7 (11.3%)	6 (9.7%)	1.11 (0.34–3.70)			
*CRP* rs1130864							
Codominant	G/G	33 (53.2%)	22 (35.5%)	1.00	0.13	169.4	0.99
	A/G	24 (38.7%)	31 (50%)	1.98 (0.90–4.34)			
	A/A	5 (8.1%)	9 (14.5%)	2.72 (0.77–9.59)			
Dominant	G/G	33 (53.2%)	22 (35.5%)	1.00	0.053	167.7	
	A/G-A/A	29 (46.8%)	40 (64.5%)	2.10 (1.00–4.45)			
Recessive	G/G-A/G	57 (91.9%)	53 (85.5%)	1.00	0.27	170.4	
	A/A	5 (8.1%)	9 (14.5%)	1.93 (0.58–6.36)			
Overdominant	G/G-A/A	38 (61.3%)	31 (50%)	1.00	0.2	170	
	A/G	24 (38.7%)	31 (50%)	1.61 (0.77–3.38)			
Log-additive	---	---	---	1.76 (1.00–3.09)	0.051	167.6	
*CRP* rs1205							
Codominant	C/C	19 (30.6%)	28 (45.2%)	1.00	0.09	168.8	0.99
	C/T	32 (51.6%)	27 (43.5%)	0.42 (0.18–0.98)			
	T/T	11 (17.7%)	7 (11.3%)	0.41 (0.13–1.30)			
Dominant	C/C	19 (30.6%)	28 (45.2%)	1.00	0.028	166.8	
	C/T-T/T	43 (69.3%)	34 (54.8%)	0.42 (0.19–0.93)			
Recessive	C/C-C/T	51 (82.3%)	55 (88.7%)	1.00	0.43	170.9	
	T/T	11 (17.7%)	7 (11.3%)	0.66 (0.23–1.87)			
Overdominant	C/C-T/T	30 (48.4%)	35 (56.5%)	1.00	0.12	169.1	
	C/T	32 (51.6%)	27 (43.5%)	0.55 (0.25–1.17)			
Log-additive	---	---	---	0.58 (0.34–1.02)	0.052	167.8	
*APOB* rs1042031							
Codominant	C/C	43 (71.7%)	42 (70%)	1.00	0.84	168.2	0.99
	C/T	16 (26.7%)	16 (26.7%)	1.14 (0.48–2.67)			
	T/T	1 (1.7%)	2 (3.3%)	1.94 (0.15–24.67)			
Dominant	C/C	43 (71.7%)	42 (70%)	1.00	0.68	166.3	
	C/T-T/T	17 (28.3%)	18 (30%)	1.19 (0.52–2.72)			
Recessive	C/C-C/T	59 (98.3%)	58 (96.7%)	1.00	0.62	166.3	
	T/T	1 (1.7%)	2 (3.3%)	1.89 (0.15–23.70)			
Overdominant	C/C-T/T	44 (73.3%)	44 (73.3%)	1.00	0.8	166.4	
	C/T	16 (26.7%)	16 (26.7%)	1.12 (0.48–2.62)			
Log-additive	---	---	---	1.21 (0.58–2.51)	0.61	166.2	
*APOB* rs6725189							
Codominant	G/G	41 (68.3%)	39 (65%)	1.00	0.81	168.1	0.56
	G/T	17 (28.3%)	18 (30%)	1.25 (0.55–2.88)			
	T/T	2 (3.3%)	3 (5%)	1.53 (0.23–10.15)			
Dominant	G/G	41 (68.3%)	39 (65%)	1.00	0.53	166.1	
	G/T-T/T	19 (31.7%)	21 (35%)	1.29 (0.58–2.84)			
Recessive	G/G-G/T	58 (96.7%)	57 (95%)	1.00	0.71	166.4	
	T/T	2 (3.3%)	3 (5%)	1.43 (0.22–9.29)			
Overdominant	G/G-T/T	43 (71.7%)	42 (70%)	1.00	0.63	166.3	
	G/T	17 (28.3%)	18 (30%)	1.22 (0.54–2.78)			
Log-additive	---	---	---	1.25 (0.64–2.42)	0.51	166.1	
*APOE* rs7412							
---	C/C	50 (80.7%)	54 (87.1%)	1.00	0.54	171.2	0.99
	C/T	12 (19.4%)	8 (12.9%)	0.73 (0.27–2.00)			
*APOE* rs429358							
---	T/T	51 (82.3%)	46 (74.2%)	1.00	0.42	170.9	0.36
	C/T	11 (17.7%)	16 (25.8%)	1.45 (0.59–3.57)			
*LIPC* rs1800588							
Codominant	C/C	38 (61.3%)	37 (60.7%)	1.00	0.27	169.6	0.44
	C/T	22 (35.5%)	18 (29.5%)	0.86 (0.39–1.92)			
	T/T	2 (3.2%)	6 (9.8%)	3.43 (0.62–19.08)			
Dominant	C/C	38 (61.3%)	37 (60.7%)	1.00	0.87	170.2	
	C/T-T/T	24 (38.7%)	24 (39.3%)	1.07 (0.50–2.26)			
Recessive	C/C-C/T	60 (96.8%)	55 (90.2%)	1.00	0.11	167.7	
	T/T	2 (3.2%)	6 (9.8%)	3.61 (0.66–19.64)			
Overdominant	C/C-T/T	40 (64.5%)	43 (70.5%)	1.00	0.52	169.8	
	C/T	22 (35.5%)	18 (29.5%)	0.77 (0.35–1.69)			
Log-additive	---	---	---	1.26 (0.69–2.30)	0.45	169.6	
*LPA* rs10455872							
---	A/A	52 (83.9%)	59 (96.7%)	1.00	0.019	165.3	0.99
	A/G	10 (16.1%)	2 (3.3%)	0.18 (0.04–0.91)			
*NOTCH1* rs13290979							
Codominant	A/A	26 (41.9%)	20 (32.8%)	1.00	0.1	168.3	0.35
	A/G	28 (45.2%)	26 (42.6%)	1.28 (0.56–2.93)			
	G/G	8 (12.9%)	15 (24.6%)	3.15 (1.05–9.46)			
Dominant	A/A	26 (41.9%)	20 (32.8%)	1.00	0.2	169.2	
	A/G-G/G	36 (58.1%)	41 (67.2%)	1.65 (0.76–3.57)			
Recessive	A/A-A/G	54 (87.1%)	46 (75.4%)	1.00	0.04	166.6	
	G/G	8 (12.9%)	15 (24.6%)	2.75 (1.02–7.43)			
Overdominant	A/A-G/G	34 (54.8%)	35 (57.4%)	1.00	0.72	170.7	
	A/G	28 (45.2%)	26 (42.6%)	0.87 (0.42–1.82)			
Log-additive	---	---	---	1.68 (0.99–2.85)	0.05	167	
*VDR* rs731236							
Codominant	A/A	32 (51.6%)	29 (47.5%)	1.00	0.54	171.6	0.67
	A/G	26 (41.9%)	24 (39.3%)	1.04 (0.48–2.27)			
	G/G	4 (6.5%)	8 (13.1%)	2.07 (0.55–7.81)			
Dominant	A/A	32 (51.6%)	29 (47.5%)	1.00	0.64	170.6	
	A/G-G/G	30 (48.4%)	32 (52.5%)	1.19 (0.57–2.48)			
Recessive	A/A-A/G	58 (93.5%)	53 (86.9%)	1.00	0.27	169.6	
	G/G	4 (6.5%)	8 (13.1%)	2.03 (0.56–7.32)			
Overdominant	A/A-G/G	36 (58.1%)	37 (60.7%)	1.00	0.85	170.8	
	A/G	26 (41.9%)	24 (39.3%)	0.93 (0.44–1.96)			
Log-additive	---	---	---	1.27 (0.73–2.22)	0.39	170.1	
*VDR* rs2228570							
Codominant	G/G	16 (25.8%)	19 (31.1%)	1.00	0.62	171.9	0.58
	A/G	36 (58.1%)	29 (47.5%)	0.70 (0.30–1.64)			
	A/A	10 (16.1%)	13 (21.3%)	1.02 (0.34–3.07)			
Dominant	G/G	16 (25.8%)	19 (31.1%)	1.00	0.53	170.4	
	A/G-A/A	46 (74.2%)	42 (68.8%)	0.77 (0.34–1.74)			
Recessive	G/G-A/G	52 (83.9%)	48 (78.7%)	1.00	0.59	170.6	
	A/A	10 (16.1%)	13 (21.3%)	1.29 (0.50–3.33)			
Overdominant	G/G-A/A	26 (41.9%)	32 (52.5%)	1.00	0.33	169.9	
	A/G	36 (58.1%)	29 (47.5%)	0.69 (0.33–1.44)			
Log-additive	---	---	---	0.97 (0.57–1.67)	0.91	170.8	
*CASR* rs1042636							
Codominant	A/A	47 (75.8%)	50 (82%)	1.00	0.25	170.1	0.08
	A/G	14 (22.6%)	8 (13.1%)	0.51 (0.19–1.38)			
	G/G	1 (1.6%)	3 (4.9%)	2.79 (0.26–29.85)			
Dominant	A/A	47 (75.8%)	50 (82%)	1.00	0.38	170.1	
	A/G-G/G	15 (24.2%)	11 (18%)	0.67 (0.27–1.65)			
Recessive	A/A-A/G	61 (98.4%)	58 (95.1%)	1.00	0.33	169.9	
	G/G	1 (1.6%)	3 (4.9%)	3.04 (0.29–32.42)			
Overdominant	A/A-G/G	48 (77.4%)	53 (86.9%)	1.00	0.16	168.9	
	A/G	14 (22.6%)	8 (13.1%)	0.50 (0.19–1.35)			
Log-additive	---	---	---	0.87 (0.42–1.80)	0.7	170.7	
*OPG* rs3134069							
Codominant	A/A	49 (79%)	52 (85.2%)	1.00	0.59	171.8	0.99
	A/C	12 (19.4%)	9 (14.8%)	0.80 (0.30–2.12)			
	C/C	1 (1.6%)	0 (0%)	0.00 (0.00–0.00)			
Dominant	A/A	49 (79%)	52 (85.2%)	1.00	0.56	170.5	
	A/C-C/C	13 (21%)	9 (14.8%)	0.75 (0.29–1.97)			
Recessive	A/A-A/C	61 (98.4%)	61 (100%)	1.00	0.35	170	
	C/C	1 (1.6%)	0 (0%)	0.00 (0.00–0.00)			
Overdominant	A/A-C/C	50 (80.7%)	52 (85.2%)	1.00	0.68	170.7	
	A/C	12 (19.4%)	9 (14.8%)	0.81 (0.31–2.16)			
Log-additive	---	---	---	0.72 (0.29–1.80)	0.48	170.3	
*OPG* rs2073618							
Codominant	G/G	15 (24.2%)	12 (19.7%)	1.00	0.85	172.5	0.07
	C/G	35 (56.5%)	37 (60.7%)	1.29 (0.51–3.27)			
	C/C	12 (19.4%)	12 (19.7%)	1.13 (0.35–3.61)			
Dominant	G/G	15 (24.2%)	12 (19.7%)	1.00	0.62	170.6	
	C/G-C/C	47 (75.8%)	49 (80.3%)	1.25 (0.51–3.08)			
Recessive	G/G-C/G	50 (80.7%)	49 (80.3%)	1.00	0.88	170.8	
	C/C	12 (19.4%)	12 (19.7%)	0.93 (0.37–2.37)			
Overdominant	G/G-C/C	27 (43.5%)	24 (39.3%)	1.00	0.6	170.6	
	C/G	35 (56.5%)	37 (60.7%)	1.22 (0.58–2.57)			
Log-additive	---	---	---	1.07 (0.60–1.91)	0.82	170.8	
*OPG* rs3102735							
Codominant	T/T	39 (62.9%)	46 (75.4%)	1.00	0.39	171	0.53
	C/T	19 (30.6%)	14 (22.9%)	0.69 (0.30–1.61)			
	C/C	4 (6.5%)	1 (1.6%)	0.29 (0.03–2.80)			
Dominant	T/T	39 (62.9%)	46 (75.4%)	1.00	0.26	169.6	
	C/T-C/C	23 (37.1%)	15 (24.6%)	0.63 (0.28–1.41)			
Recessive	T/T-C/T	58 (93.5%)	60 (98.4%)	1.00	0.28	169.7	
	C/C	4 (6.5%)	1 (1.6%)	0.32 (0.03–3.08)			
Overdominant	T/T-C/C	43 (69.3%)	47 (77%)	1.00	0.48	170.3	
	C/T	19 (30.6%)	14 (22.9%)	0.74 (0.32–1.70)			
Log-additive	---	---	---	0.63 (0.32–1.26)	0.19	169.1	
*CALCR* rs1801197							
Codominant	A/A	32 (51.6%)	37 (60.7%)	1.00	0.48	171.4	0.99
	A/G	27 (43.5%)	20 (32.8%)	0.64 (0.29–1.39)			
	G/G	3 (4.8%)	4 (6.6%)	1.19 (0.24–5.98)			
Dominant	A/A	32 (51.6%)	37 (60.7%)	1.00	0.34	169.9	
	A/G-G/G	30 (48.4%)	24 (39.3%)	0.69 (0.33–1.46)			
Recessive	A/A-A/G	59 (95.2%)	57 (93.4%)	1.00	0.65	170.6	
	G/G	3 (4.8%)	4 (6.6%)	1.43 (0.29–6.99)			
Overdominant	A/A-G/G	35 (56.5%)	41 (67.2%)	1.00	0.23	169.4	
	A/G	27 (43.5%)	20 (32.8%)	0.63 (0.29–1.35)			
Log-additive	---	---	---	0.82 (0.45–1.52)	0.54	170.5	
*F2* rs1799963							
---	G/G	59 (98.3%)	58 (98.3%)	1.00	0.77	164.3	0.99
	A/G	1 (1.7%)	1 (1.7%)	0.64 (0.04–11.58)			
*F5* rs6025							
---	C/C	55 (91.7%)	57 (96.6%)	1.00	0.18	162.5	0.99
	C/T	5 (8.3%)	2 (3.4%)	0.31 (0.05–1.84)			
*F5* rs6027							
Codominant	T/T	47 (78.3%)	46 (78%)	1.00	0.92	166.2	0.09
	C/T	11 (18.3%)	11 (18.6%)	0.86 (0.33–2.29)			
	C/C	2 (3.3%)	2 (3.4%)	1.33 (0.17–10.31)			
Dominant	T/T	47 (78.3%)	46 (78%)	1.00	0.87	164.3	
	C/T-C/C	13 (21.7%)	13 (22%)	0.92 (0.37–2.29)			
Recessive	T/T-C/T	58 (96.7%)	57 (96.6%)	1.00	0.77	164.3	
	C/C	2 (3.3%)	2 (3.4%)	1.36 (0.18–10.49)			
Overdominant	T/T-C/C	49 (81.7%)	48 (81.4%)	1.00	0.75	164.3	
	C/T	11 (18.3%)	11 (18.6%)	0.85 (0.32–2.25)			
Log-additive	---	---	---	0.99 (0.47–2.07)	0.97	164.4	
*F7* rs6046							
Codominant	G/G	52 (86.7%)	42 (71.2%)	1.00	0.15	162.5	0.20
	A/G	7 (11.7%)	15 (25.4%)	2.55 (0.91–7.16)			
	A/A	1 (1.7%)	2 (3.4%)	2.94 (0.25–35.06)			
Dominant	G/G	52 (86.7%)	42 (71.2%)	1.00	0.052	160.5	
	A/G-A/A	8 (13.3%)	17 (28.8%)	2.59 (0.98–6.90)			
Recessive	G/G-A/G	59 (98.3%)	57 (96.6%)	1.00	0.46	163.8	
	A/A	1 (1.7%)	2 (3.4%)	2.48 (0.21–29.33)			
Overdominant	G/G-A/A	53 (88.3%)	44 (74.6%)	1.00	0.08	161.3	
	A/G	7 (11.7%)	15 (25.4%)	2.45 (0.88–6.87)			
Log-additive	---	---	---	2.19 (0.94–5.11)	0.058	160.8	
*F13A1* rs5985							
Codominant	C/C	39 (65%)	37 (62.7%)	1.00	0.33	164.1	0.10
	A/C	15 (25%)	19 (32.2%)	1.74 (0.72–4.21)			
	A/A	6 (10%)	3 (5.1%)	0.66 (0.15–2.91)			
Dominant	C/C	39 (65%)	37 (62.7%)	1.00	0.4	163.7	
	A/C-A/A	21 (35%)	22 (37.3%)	1.41 (0.63–3.14)			
Recessive	C/C-A/C	54 (90%)	56 (94.9%)	1.00	0.41	163.7	
	A/A	6 (10%)	3 (5.1%)	0.55 (0.13–2.37)			
Overdominant	C/C-A/A	45 (75%)	40 (67.8%)	1.00	0.17	162.5	
	A/C	15 (25%)	19 (32.2%)	1.83 (0.77–4.36)			
Log-additive	---	---	---	1.09 (0.60–1.98)	0.78	164.3	
*ITGB3* rs5918							
Codominant	T/T	45 (75%)	42 (71.2%)	1.00	0.95	166.3	0.08
	C/T	12 (20%)	14 (23.7%)	1.11 (0.45–2.77)			
	C/C	3 (5%)	3 (5.1%)	0.83 (0.15–4.65)			
Dominant	T/T	45 (75%)	42 (71.2%)	1.00	0.9	164.4	
	C/T-C/C	15 (25%)	17 (28.8%)	1.05 (0.45–2.46)			
Recessive	T/T-C/T	57 (95%)	56 (94.9%)	1.00	0.81	164.3	
	C/C	3 (5%)	3 (5.1%)	0.81 (0.15–4.46)			
Overdominant	T/T-C/C	48 (80%)	45 (76.3%)	1.00	0.8	164.3	
	C/T	12 (20%)	14 (23.7%)	1.13 (0.46–2.79)			
Log-additive	---	---	---	1.00 (0.51–1.95)	1	164.4	

Here and below: TLR is for Toll-like receptor, TREM is for triggering receptor expressed on myeloid cells, IL is for interleukin, TNF is for tumor necrosis factor, CRP is for C-reactive protein, APO is for apolipoprotein, LIPC is for hepatic lipase, LPA is for lipoprotein (a), VDR is for vitamin D receptor, CASR is for calcium-sensing receptor, OPG is for osteoprotegerin, CALCR is for calcitonin receptor, ITGB is for integrin beta, OR is for odds ratio, CI is for confidence interval, AIC is for Akaike information criterion, and HWE is for Hardy–Weinberg equilibrium.

**Table 2:** Brief description of the model predicting the risk of severe bioprosthetic mitral valve calcification after mitral valve replacement surgery, calculated by stepwise logistic regression.

**Clinical Markers**	
Gender	Male gender OR = 2.80 (95% CI = 1.23–6.38)
Age	No statistically significant association
Coronary artery disease	No statistically significant association
Peripheral artery disease	No statistically significant association
Arterial hypertension	No statistically significant association
Diabetes mellitus	No statistically significant association
**Genomic Markers**	
rs3775073 (*TLR6*)	Carriers of T/T genotype: OR = 3.33 (95% CI = 1.14–9.75)
rs2229238 (*IL6R*)	Carriers of C/T genotype: OR = 3.70 (95% CI = 1.48–9.22)
rs10455872 (*LPA*)	Carriers of A/A genotype: OR = 5.67 (95% CI = 1.19–27.09)
rs5743810 (*TLR6*)	No statistically significant association
rs1800871 (*IL10*)	No statistically significant association
rs1800872 (*IL10*)	No statistically significant association
rs1205 (*CRP*)	No statistically significant association
rs13290979 (*NOTCH1*)	No statistically significant association
**General Evaluation**	
Sensitivity	59.68% (37 true; 25 false-negatives)
Specificity	74.19% (46 true; 16 false-positives)
Percent of cases correctly classified	66.94%
Area under the ROC curve	0.73 (95% CI = 0.64–0.81)
Standard error	0.045

Here and below: ROC is for receiver operating characteristic.

**Table 3:** Clinical features of the patients who underwent mitral valve replacement surgery.

Feature	Value, *n* (%)
Male gender	50 (40.32%)
Age ≥ 50 years	65 (52.42%)
Mitral stenosis and/or regurgitation with New York Heart Association functional class III-IV symptoms	54 (43.55%)
Coronary artery disease	14 (11.29%)
Peripheral artery disease	6 (4.84%)
Arterial hypertension	38 (30.64%)
Diabetes mellitus	8 (6.45%)
Severe bioprosthetic mitral valve calcification within 8 years post-implantation	62 (50.00%)

**Table 4:** Basic and echocardiography characteristics of the study population.

Feature	Without Severe Bioprosthetic Mitral Valve Calcification	With Severe Bioprosthetic Mitral Valve Calcification	Total	*p* Value
Basic characteristics				
Sample size	62 (50.00%)	62 (50.00%)	124 (100.00%)	
Mean age	50.60 (48.12–53.08)	47.81 (45.68–49.94)	49.20 (47.57–50.83)	0.09
Standard deviation of mean age	9.76	8.39	9.17	
Male gender	19 (30.64%)	31 (50.00%)	50 (40.32%)	0.03
Female gender	43 (69.36%)	31 (50.00%)	74 (59.68%)	
Echocardiography characteristics				
Left atrial diameter, cm	6.70 (6.43–7.01)	5.51 (5.22–5.69)	6.10 (5.82–6.35)	0.02
Left ventricular end-diastolic diameter, cm	5.42 (5.23–5.56)	5.37 (5.17–5.50)	5.39 (5.20–5.53)	0.81
Left ventricular end-systolic diameter, cm	3.23 (3.05–3.39)	3.41 (3.26–3.51)	3.32 (3.15–3.45)	0.36
Left ventricular end-diastolic volume, cm^3^	139.03 (136.12–143.15)	136.56 (134.01–139.76)	137.79 (135.06–141.45)	0.82
Left ventricular end-systolic volume, cm^3^	40.23 (38.23–41.98)	45.14 (43.24–47.12)	42.68 (40.73–44.55)	0.03
Interventricular septal thickness, cm	1.04 (0.97–1.12)	1.08 (1.02–1.15)	1.06 (0.99–1.13)	0.89
Left ventricular posterior wall thickness, cm	1.03 (0.95–1.08)	1.11 (1.00–1.18)	1.07 (0.97–1.13)	0.72
Left ventricular ejection fraction, %	71.00 (67.00–74.00)	65.00 (61.00–68.00)	68.00 (64.00–71.00)	0.03
Right atrial diameter, cm	6.00 (5.87–6.16)	4.70 (4.62–4.88)	5.35 (5.24–5.52)	0.03
Right ventricular diameter, cm	2.09 (2.01–2.17)	2.03 (1.95–2.14)	2.06 (1.98–2.15)	0.76
Aortic root diameter, cm	3.30 (3.12–3.49)	3.32 (3.14–3.50)	3.31 (3.13–3.49)	0.93
Mitral valve area, cm^2^	1.72 (1.64–1.79)	1.41 (1.35–1.47)	1.56 (1.49–1.63)	0.02

**Table 5:** Features of the genotyped polymorphisms.

Single Nucleotide Polymorphism	Nucleotide Substitution	Chromosomal Position	Amino Acid Substitution	Forward 5′-3′ and Reverse 3′-5′ Polymerase Chain Reaction Primers
*TLR1* gene				
rs5743551	T>C	38807654	5′-upstream	F: agtgggcagggcagtaagggaagct R: ctcagcactctgaattcctgttttt
rs5743611	C>G	38800214	Arg80Thr	F: aacactgatatcaagatactggatt R: tattatgagaaattatcaaaatcct
*TLR2* gene				
rs3804099	T>C	154624656	Asn199Asn	F: caaaaagtttgaagtcaattcagaa R: gtaagtcatctgatccttcatatga
rs5743708	G>A	154626317	Arg753Gln	F: aagccattccccagcgcttctgcaagctgc R: gaagataatgaacaccaagacctacctgga
*TLR4* gene				
rs4986790	A>G	120475302	Asp299Gly	F: gattagcatacttagactactacctcgatg R: attattgacttatttaattgtttgacaaat
rs4986791	C>T	120475602	Thr399Ile	F: gttgctgttctcaaagtgattttgggacaa R: agcctaaagtatttagatctgagcttcaat
*TLR6* gene				
rs3775073	T>C	38829832	Lys421Lys	F: cactatactctcaacccaagtgcagttttc R: ttatgtctaccagattccaaagaattccagc
rs5743810	A>G	38830350	Ser249Pro	F: ttgagggtaaaattcagtaaggttg R: acctctggtgagttctgataaaaat
*TREM-1* gene				
rs1817537	C>G	41244567	intronic	F: acacagggacagacagatggcaatggaaca R: aaggccagatgcagagccagtgctatgcag
rs3804277	C>T	41245172	intronic	F: ccagcatctctctcacccctcacatggtgg R: cactcagcatcctcagcatctgccccgatt
rs6910730	A>G	41246633	3′-downstream	F: catggagcaacaccaaggtctaggggcaag R: aatctaggatggattcgtgctgacttccca
rs7768162	A>G	41255511	5′-upstream	F: aaagattcctactgctaaataaacaaaaaa R: taacttggtttcttcaaaggaattgaaata
rs2234246	C>T	41243740	3′-UTR	F: ggaaggtgagacgctgactttagaaatagc R: ggtgattacagatttaattcatgttattaa
rs4711668	T>C	41246473	3′-downstream	F: gctagtgtggattccactttccagactgga R: ttggctgaaaggatagttcatattagatga
rs9471535	T>C	41255490	5’-upstream	F: aaaatttttaaatttaaataaaaagattcc R: ctgctaaataaacaaaaaaataacttggtt
rs2234237	T>A	41250466	Thr25Ser	F: gcccctctttcagttcatacttttcctcag R: aatttagttgcagctcggagttctataagc
*IL1B* gene				
rs16944	A>G	113594867	5′-upstream	F: taccttgggtgctgttctctgcctc R: ggagctctctgtcaattgcaggagc
rs1143634	G>A	113590390	Phe105Phe	F: cataagcctcgttatcccatgtgtc R: aagaagataggttctgaaatgtgga
*IL1F9* gene				
rs17659543	C>T	113716306	Not announced	F: tgtacctggacaagaggcataaattggggc R: gtcttaggaaagcagatatacagccatcct
*IL6* gene				
rs1554606	T>G	22768707	intronic	F: ttagttcatcctgggaaaggtactc R: cagggccttttccctctctggctgc
rs1800796	G>C	22766246	5′-upstream	F: atggccaggcagttctacaacagcc R: ctcacagggagagccagaacacaga
rs2069827	G>T	22765456	5′-upstream	F: gcccaacagaggtcactgttttatc R: atcttgaagagatctcttcttagca
*IL6R* gene				
rs2228145	A>T/C	154426970	Asp358Val/Ala	F: aattttttttttaacctagtgcaag R: ttcttcttcagtaccactgcccaca
rs2229238	T>C	154437896	3′-UTR	F: ccagcagcctggaccctgtggatga R: aaaacacaaacgggctcagcaaaag
*IL8* gene				
rs2227306	C>T	74607055	intronic	F: aactctaactctttatataggaagt R: gttcaatgttgtcagttatgactgt
*IL10* gene				
rs1800871	A>G	206946634	5′-upstream	F: agtgagcaaactgaggcacagagat R: ttacatcacctgtacaagggtacac
rs1800872	T>G	206946407	5’-upstream	F: ttttactttccagagactggcttcctacag R: acaggcggggtcacaggatgtgttccaggc
rs1800896	T>C	206946897	5′-upstream	F: tcctcttacctatccctacttcccc R: tcccaaagaagccttagtagtgttg
*IL12B* gene				
rs3212227	T>G	158742950	3′-UTR	F: attgtttcaatgagcatttagcatc R: aactatacaaatacagcaaagatat
*IL12RB* gene				
rs375947	A>G	18180451	Met365Thr	F: aggctgccattcaatgcaatacgtc R: tgctctgagcccgggctggccaata
*TNF* gene				
rs361525	G>A	31543101	5′-upstream	F: ggcccagaagacccccctcggaatc R: gagcagggaggatggggagtgtgag
rs1800629	G>A	31543031	5′-upstream	F: gaggcaataggttttgaggggcatg R: ggacggggttcagcctccagggtcc
rs1799964	T>C	31542308	3′-downstream	F: gcaggggaagcaaaggagaagctgagaaga R: gaaggaaaagtcagggtctggaggggcggg
*CRP* gene				
rs3093077	A>C	159679636	Not announced	F: ggaatccaggcaagtacgacaaccc R: tctgagactagtgggcagttgtcct
rs1130864	G>A	159683091	3′-UTR	F: cctcaaattctgattcttttggacc R: tttcccagcatagttaacgagctcc
rs1205	C>T	159682233	3′-UTR	F: acttccagtttggcttctgtcctca R: agtctctctccatgtggcaaacaag
*APOB* gene				
rs1042031	C>T	21225753	Glu4181Lys	F: caatcagatgcttgactttcatatggaatt R: ttgagtaactcgtaccaagccatcaaacac
rs6725189	G>T	21219001	Not announced	F: ttcccagcctcagctcaacagagctatggg R: cagcagtcggccctctctattgttctttcc
*APOE* gene				
rs7412	C>T	45412079	Arg176Cys	F: ctcctccgcgatgccgatgacctgcagaag R: gcctggcagtgtaccaggccggggcccgcg
rs429358	T>C	45411941	Cys130Arg	F: gcccggctgggcgcggacatggaggacgtg R: gcggccgcctggtgcagtaccgcggcgagg
*LIPC* gene				
rs1800588	C>T	58723675	5′-upstream	F: tctttgcttcttcgtcagctccttttgaca R: gggggtgaagggttttctgcaccacacttt
*LPA* gene				
rs10455872	A>G	161010118	intronic	F: tcagacaccttgttctcagaaccca R: tgtgtttatacaggttagaggagaa
*NOTCH1* gene				
rs13290979	A>G	139425634	intronic	F: ccagcccagcagtgaagaaactgagcccac R: accctcctggcctgacctacactcgggctt
*VDR* gene				
rs731236	A>G	48238757	Ile352Ile	F: tgtgttggacaggcggtcctggatggcctc R: atcagcgcggcgtcctgcaccccaggacga
rs2228570	A>G	48272895	Met1Thr/Lys/Arg	F: ggcagggaagtgctggccgccattgcctcc R: tccctgtaagaacagcaagcaggccacggt
*CASR* gene				
rs1042636	A>G	122003769	Arg990Gly	F: gatgagcctcagaagaacgccatggcccac R: ggaattctacgcaccagaactccctggagg
*OPG* gene				
rs3134069	A>C	119964988	5′-upstream	F: ggagcttcctacgcgctgaacttctggagt R: gcctcctcgaggtctttccactagcctcaa
rs2073618	G>C	119964052	Asn3Lys	F: gggacttaccacgagcgcgcagcacagcaa R: ttgttcattgtggtccccggaaacctcagg
rs3102735	T>C	119965070	5′-upstream	F: ctttgctctagggttcgctgtctcccccat R: aattccctggtctagaagttagacttgatg
*CALCR* gene				
rs1801197	A>G	93055753	Leu481Pro	F: tcgccttggttgttggctggttcattcctc R: gctcctgatggcagatgtaaattgggatgt
*F2* gene				
rs1799963	G>A	46761055	3′-UTR	F: gttcccaataaaagtgactctcagc R: agcctcaatgctcccagtgctattc
*F5* gene				
rs6025	T>C	169519049	Gln534Arg	F: ttacttcaaggacaaaatacctgtattcct R: gcctgtccagggatctgctcttacagatta
rs6027	T>C	169483561	Asp2222Gly	F: gggtttttgaatgttcaattctagtaaata R: cacagccaaagagttccaggcgaagtgcaa
*F7* gene				
rs6046	G>A	113773159	Arg412Gln/Pro/Leu	F: acagtggaggcccacatgccacccactacc R: gggcacgtggtacctgacgggcatcgtcag
*F13A1* gene				
rs5985	C>A	6318795	Val35Leu	F: taccttgcaggttgacgccccggggcacca R: gccctgaagctccactgtgggcaggtcatc
*ITGB3* gene				
rs5918	T>C	45360730	Leu59Pro	F: tttgggctcctgacttacaggccctgcctc R: gggctcacctcgctgtgacctgaaggagaa
